# Trends in hospitalizations of children with respiratory syncytial virus aged less than 1 year in Italy, from 2015 to 2019

**DOI:** 10.1186/s13052-024-01688-9

**Published:** 2024-06-21

**Authors:** Renato Cutrera, Daniela d’Angela, Massimiliano Orso, Liliana Guadagni, Anna Chiara Vittucci, Ilaria Bertoldi, Barbara Polistena, Federico Spandonaro, Ciro Carrieri, Eva Agostina Montuori, Raffaella Iantomasi, Luigi Orfeo

**Affiliations:** 1 Pediatric Pulmonology & Cystic Fibrosis Unit, Respiratory Research Unit, Bambino Gesù Children’s Hospital, IRCCS, Rome, Italy; 2 C.R.E.A. Sanità (Centre for Applied Economic Research in Healthcare), Rome, Italy; 3 University of Rome Tor Vergata, Rome, Italy; 4 Department of Surgical and Biomedical Sciences, University of Perugia, Perugia, Italy; 5 Hospital University Pediatrics Clinical Area, Bambino Gesù Children’s Hospital IRCCS, Rome, Italy; 6 Vaccine Medical Department, Pfizer, Rome, Italy; 7 Neonatal Intensive Care Unit, Ospedale Isola Tiberina Gemelli Isola, Rome, Italy

**Keywords:** Respiratory syncytial virus, RSV, Infants, Hospitalization, Burden, Italy, Hospital discharge record

## Abstract

**Background:**

Respiratory syncytial virus (RSV) affects 60–80% of children below 1 year and it’s the first cause of acute bronchiolitis. The aim of this study was to assess the trend and characteristics of hospitalizations for RSV infections in Italy.

**Methods:**

This is a retrospective study based on the Italian Hospital Discharge Record (HDR) database. We analysed HDRs from June 2015 to May 2019, considering two groups of infants: Group 1 had a confirmed diagnosis of RSV; Group 2 had a diagnosis of acute bronchiolitis not RSV-coded.

**Results:**

There were 67,746 overall hospitalizations (40.1% Group 1, and 59.9% Group 2). Hospitalization rate increased for Group 1 from 125 to 178 per 10,000 infants (+ 42.4%), and for Group 2 from 210 to 234 per 10,000 (+ 11.4%). The mean hospitalization length was 6.3 days in Group 1, longer than Group 2 (+ 1.0 day). A further analysis revealed that infants with heart disease or born premature had longer mean hospital stay compared to infants without risk factors (10.7 days versus 6.1 days, *p* < 0.0001; 34.0 days versus 6.1 days, *p* < 0.0001, respectively). Group 1 required more critical care (oxygen therapy and/or mechanical ventilation) than Group 2. We found that, in proportion to hospital admissions in pediatric and general hospitals, RSV was more frequently diagnosed in the first ones. The mean hospitalization cost increased for Group 1 (from € 2,483 to € 2,617) and Group 2 (from € 2,007 to € 2,180).

**Conclusions:**

Our results confirmed that RSV pulmonary disease in infants is seasonal and often requires hospitalization. Our study suggested that RSV is responsible for an increasing hospitalization rate and related costs during the study period.

**Supplementary Information:**

The online version contains supplementary material available at 10.1186/s13052-024-01688-9.

## Introduction

Respiratory syncytial virus (RSV) is one of the most common respiratory infections in children below the age of 5 [1]. RSV infection affects 60–80% of children by the age of 1 year, and almost all children by the age of 2 years. It’s the most common cause of pediatric hospitalization [2], representing a major socioeconomic worldwide burden [3]. RSV is also the first cause of death among respiratory infections in children under 1 year of life [4–6]. RSV belongs to the Pneumovirus genus and is a medium-sized enveloped RNA Paramyxovirus and only infects humans. RSV infections exhibit clear seasonality in temperate areas, beginning in late fall or early winter, peaking between mid-December and early February, and ending in late spring [7]. The clinical symptoms can range from minor upper respiratory tract infection (URTI) or otitis media to severe and potentially fatal lower respiratory tract infection (LRTI). Bronchiolitis is the most typical LRTI in infants with RSV infection [7]. A UK study reported that around 36% of general practitioner episodes of care for RSV-attributable respiratory disease occurred in children 2–4 years of age, with an acute bronchitis and bronchiolitis rate of 6.3% (5–17 years old) and an otitis media rate of 13.0% (2–4 years old) [8].

Currently, there are very few available options to prevent or treat RSV [1]. In recent years, two drugs have been authorized by the European Medicines Agency (EMA): Synagis (palivizumab) and Beyfortus (nirsevimab). Palivizumab is a recombinant humanised monoclonal antibody produced by DNA technology, indicated for the prevention of serious LRTI requiring hospitalization caused by RSV in children at high risk for RSV disease [9]. Nirsevimab is a human immunoglobulin G1 kappa monoclonal antibody produced by recombinant DNA technology, indicated for the prevention of RSV LRTI in neonates and infants during their first RSV season [10]. To deeply analyze safety and tolerability, nirsevimab has been maintained under additional monitoring by EMA [1]. Several other monoclonal antibodies, vaccines, and antivirals have been investigated in clinical trials [1]. The global burden of RSV calls for the development of effective preventive and therapeutic pharmacological measures. In order to plan prevention strategies, it is important to investigate the epidemiology of RSV in the pediatric population. The aim of this study was to assess the trend and characteristics of hospitalizations due to RSV infections in Italian infants below 1 year of age between June 2015 and May 2019.

## Methods

This is a retrospective study based on the Hospital Discharge Record (HDR) database held by the Italian Ministry of Health, reported following the RECORD (The REporting of studies Conducted using Observational Routinely-collected health Data) statement [11]. Ethical approval and patients’ informed consent for this study were not required because only anonymized data were used. HDRs routinely collect demographic and clinical data of all patients admitted to Italian public and private hospitals. Clinical data are coded using the ICD-9-CM coding system (International Classification of Disease, 9th Revision, Clinical Modification). Each HDR reports the primary diagnosis and up to 5 secondary diagnoses, along with surgical interventions, diagnostic and therapeutic procedures performed during hospital stay. For this study, we included children aged less than 1 year having a primary or secondary diagnosis in the following two groups:

- Group 1: ICD-9 codes 079.6 (Respiratory syncytial virus), 466.11 (Acute bronchiolitis due to respiratory syncytial virus), 480.1 (Pneumonia due to respiratory syncytial virus).
- Group 2: ICD-9 code 466.19 (Acute bronchiolitis due to other infectious organisms).

Then we analyzed the relative HDRs for the following seasons: (1) June 2015 – May 2016; (2) June 2016 – May 2017; (3) June 2017 – May 2018; (4) June 2018 – May 2019. We considered children in Group 1 having a confirmed diagnosis of RSV, obtained by a diagnostic test such as rapid antigen test or antigen detection by direct or indirect immunofluorescence or ELISA. As for Group 2, we hypothesized that it collects most of children having RSV without coding, due to etiology of acute bronchiolitis of which RSV accounts for 60–80% of cases [12].

For each season, we gathered and analyzed the following variables: number of hospitalizations, hospitalization rate, mortality rate, mean length of hospital stay, type of healthcare facility (pediatric vs. general), cost of hospitalization. Additionally, we investigated comorbidities to verify whether they could influence length of hospital stay and deaths. All variables are referred to the entire Italian territory, except hospitalization rate that had also a focus on geographical areas due to different climates.

### Statistical analysis

Descriptive and analytic statistics were reported. Discrete data were reported as number and percentages, while continuous data as means and standard deviations with 95% confidence interval (CI). The mean length of hospital stay between Groups was compared by the unpaired two-sided t-test. A multivariable linear regression model was used to determine the relationship between the length of hospital stay and various comorbidities. P-values less than 0.05 were considered statistically significant.

Hospitalization rate was computed as the number of hospital admissions per year divided by the resident infant population in the same period, as reported by the Italian National Statistics Institute (Istat) [13]. Cost of hospitalization, associated to all considered admissions with a diagnosis in primary or secondary position, was calculated using the rates of the Italian national cost nomenclator (Diagnosis Related Groups – DRG) [14]. A list of DRG considered in season 4 is reported in the Additional file 1.

Data were analyzed through the Microsoft Excel 2016 [15] and STATA 13 [16] software.

## Results

**Fig. 1:**
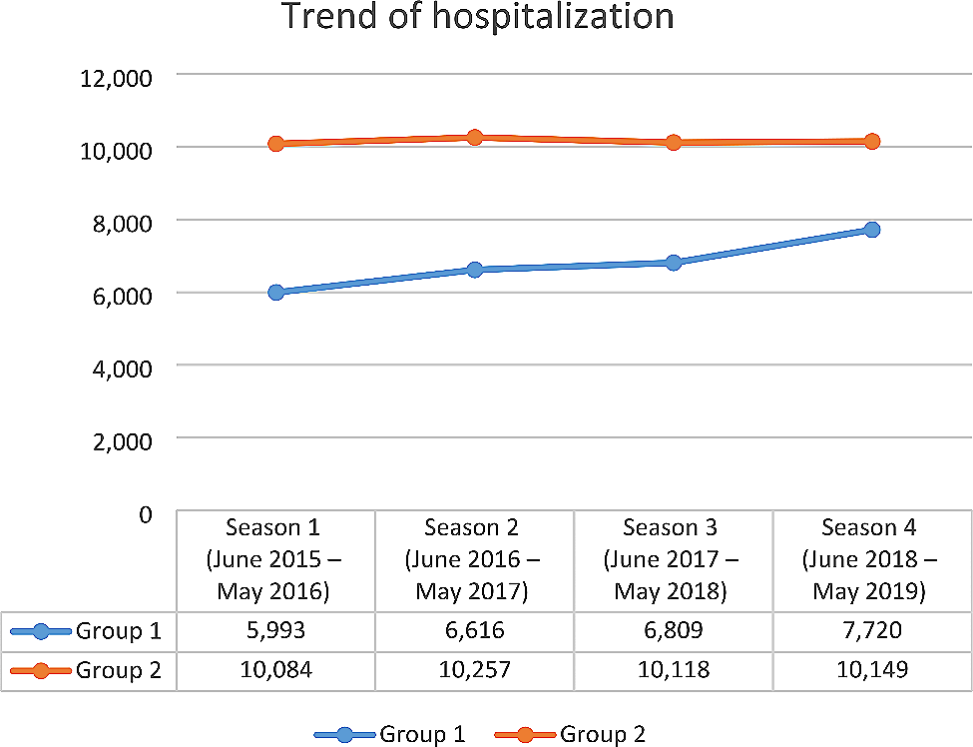
Trend of hospitalizations in Italy

In the study period (June 2015-May 2019) there were 67,746 overall hospitalizations, of which 40.1% were in Group 1 (RSV-coded), and 59.9% in Group 2 (not RSV-coded). As Fig. 1 shows, the trend of hospitalizations increased for Group 1 from season 1 to season 4 (+ 28.8%), while the number of hospitalizations for Group 2 was stable through the years.

**Fig. 2:**
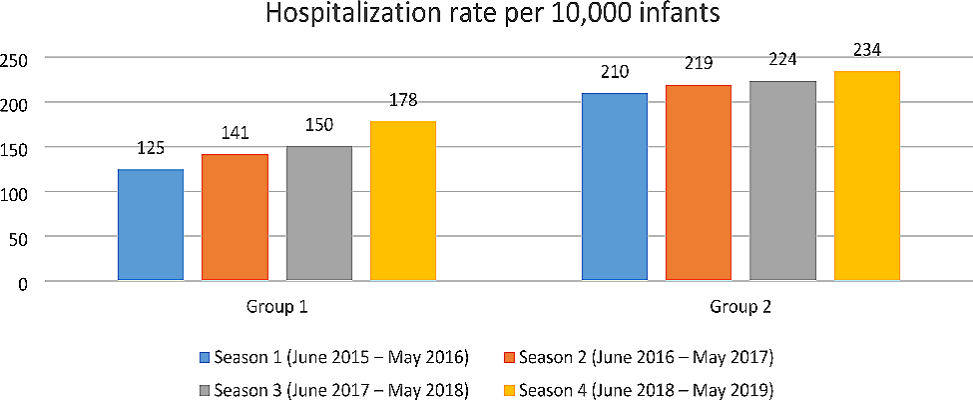
Trend of hospitalization rate in Italy

As for hospitalization rate, we can observe a higher increase for Group 1 (+ 42.4%) than for Group 2 (+ 11.4%) (Fig. 2).

**Fig. 3:**
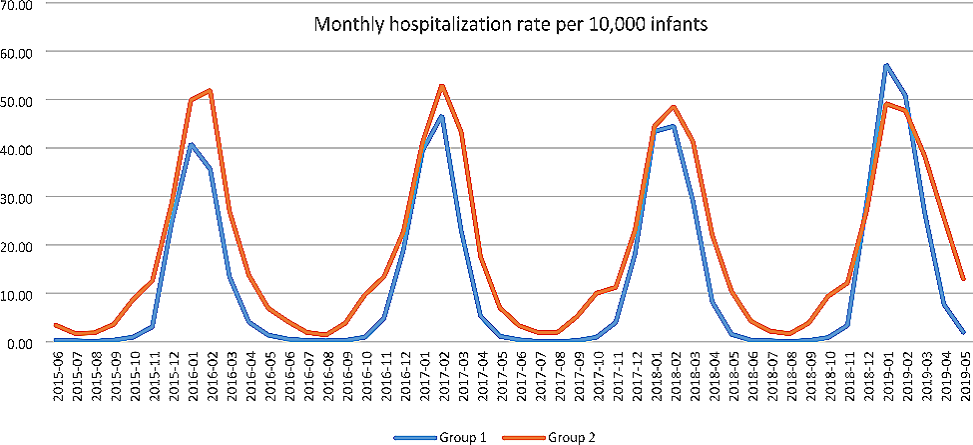
Monthly hospitalization rate per 10,000 infants from season 1 to season 4

In Fig. 3 we analysed the monthly trend for all seasons. The peak seasonality for both groups was between January and February, and in the remaining months Group 2 had a higher hospitalization rate than Group 1. Comparing season 1 and season 4, we noticed that Group 1 surpassed Group 2 in hospitalization rate during the peak.

**Fig. 4:**
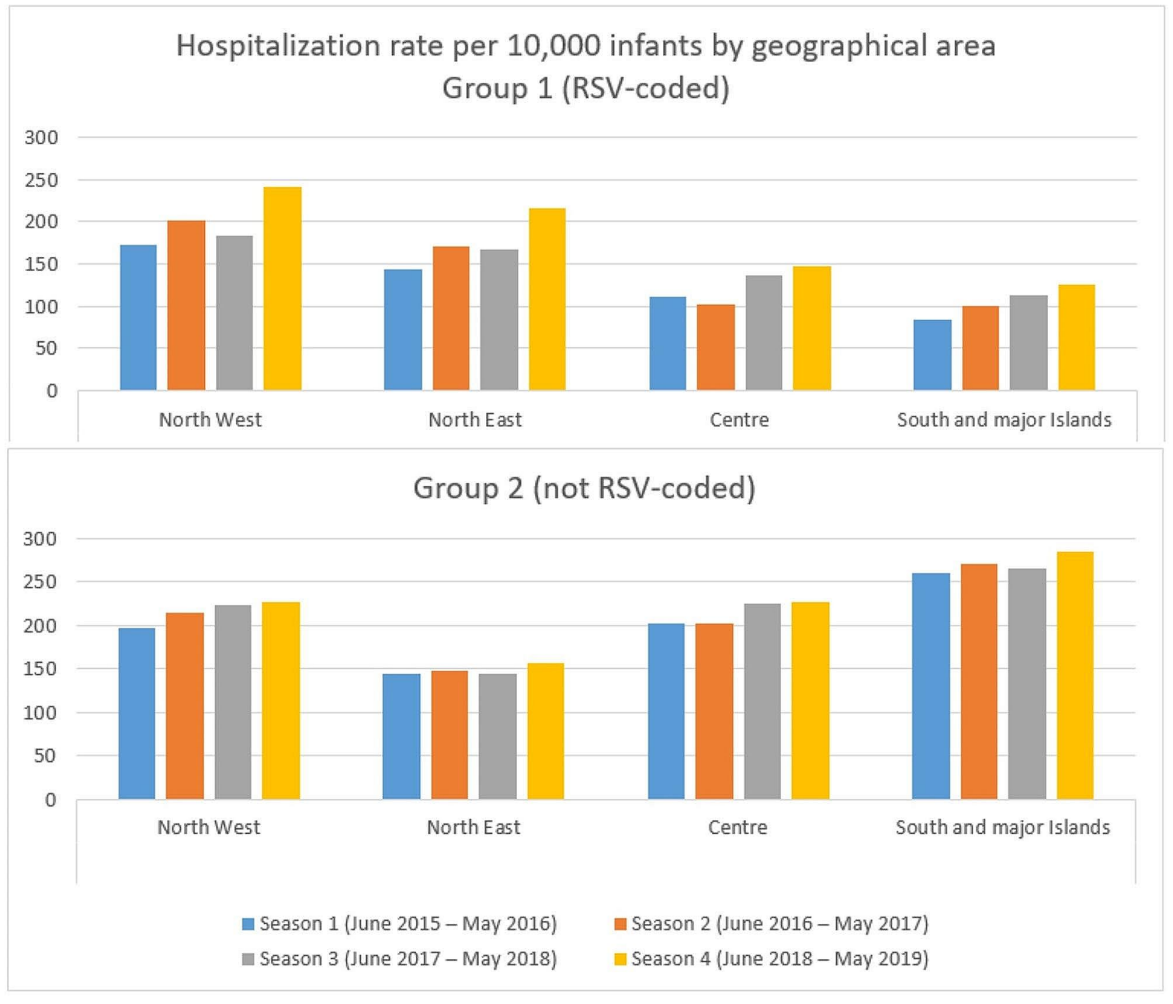
Hospitalization rate per 10,000 infants by geographical area: Group 1 (RSV-coded) and Group 2 (not RSV-coded)

Furthermore, we analyzed hospitalization rates by geographical area (North West, North East, Centre, South and major Islands). In Group 1, we noticed an increase in hospitalization rate for every area, with some variations between seasons. In particular, from season 1 to season 4 the North East had the highest increase (+ 50.7%), followed by South and major Islands (+ 47.9%), North West (+ 40.0%), and Centre (+ 32.9%). Conversely, in Group 2 we observed a smaller increase: North West (+ 14.7%), North East (+ 9.2%), Centre (+ 12.9%), South and major Islands (+ 10.0%) (Fig. 4).

As for in-hospital mortality, we recorded 19 total deaths out of 67,746 hospitalizations in the study period. The mortality rate in the four seasons ranged from 0.13 to 0.33/1,000 per year in Group 1 and from 0.19 to 0.40/1,000 per year in Group 2. Out of 7 deaths in Group 1, 2 deaths were not caused by RSV: one infant suffered from heart disease, and another had cardiac arrest. Instead, out of 12 deaths occurred in Group 2, 9 were not retained to be caused primarily by bronchiolitis (heart diseases, sepsis, bone marrow transplant rejection, meningitis).

**Fig. 5:**
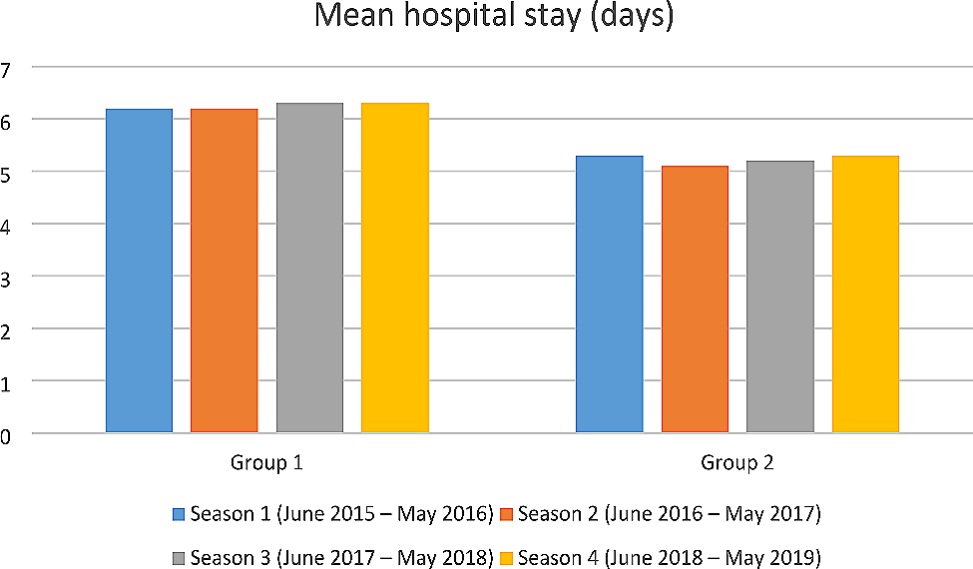
Mean hospital stay (days)

We analysed the mean hospitalization length for the two groups and the emerging data was the significantly longer hospital stay in Group 1, compared to Group 2 (+ 1.04 day; 95% CI, 0.96–1.11; *p* < 0.0001) (Fig. 5). However, in each group the hospital stay length was very similar between seasons.

**Fig. 6:**
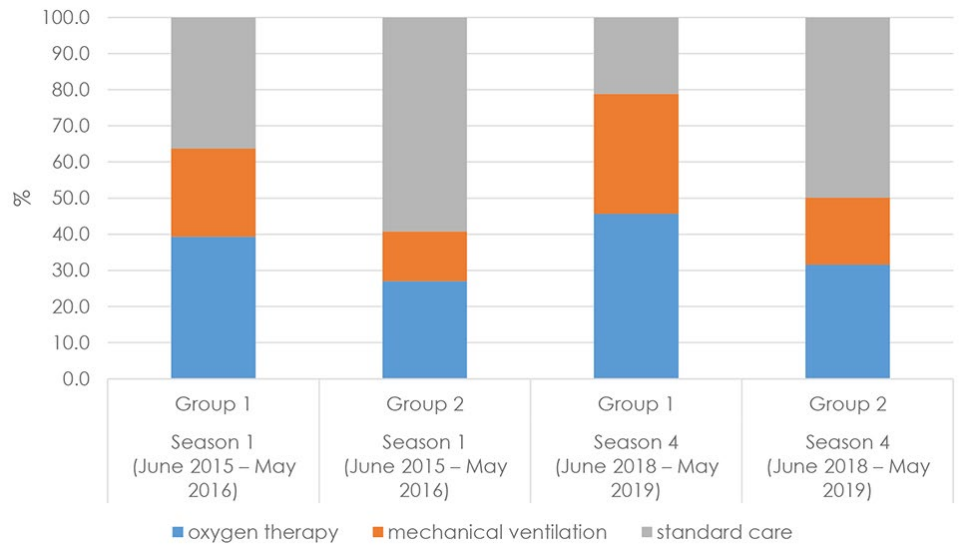
Rates of critical care cases out of all hospitalizations

Out of all hospitalizations, Group 1 required more critical care (oxygen therapy and/or mechanical ventilation) than Group 2 (+ 23.0% and + 28.7% in season 1 and in season 4, respectively), although throughout the seasons we observed an increment of critical care in both groups (Fig. 6).

In order to investigate comorbidities, we divided Group 1 into three subgroups: (A) infants with heart disease; (B) premature infants; (C) infants without risk factors. Then, we analyzed the mean length of hospital stay in each subgroup. We also verified whether the number of comorbidities could have an impact on the length of hospital stay. The mean hospital stay of subgroup A and subgroup B were significantly longer than that of subgroup C (10.7 days versus 6.1 days, *p* < 0.0001; 34.0 days versus 6.1 days, *p* < 0.0001). In each subgroup, we noticed that the higher the number of diagnoses, the longer the length of hospital stay.

**Table:** 

	Heart disease	Premature infants	No risk factors	Total
Number of hospitalizations	412	56	26,687	27,155
Mean length of hospital stay	10.7	34.0	6.1	6.2

Furthermore, the multivariable linear regression model yielded a statistically significant result, as indicated by an F-statistic of 478.85 (*p* < 0.0001). Specifically, the following variables demonstrated concurrent statistical significance within the model and were associated with longer hospital stay: prematurity, cytomegalovirus infection, sepsis, Down syndrome, pertussis, esophageal reflux, heart disease, rotavirus gastroenteritis, Hemophilus influenzae infection, urinary tract infection, anemia, rhinovirus, and belonging to Group 1 (RSV-coded). All variables except one (rhinovirus, *p* = 0.013) had a p-value < 0.001.

Considering the type of healthcare facility (pediatric vs. general hospitals), we found that in season 4 infants in Group 1 (RSV-coded) admitted in pediatric hospitals were 7.2% of the total (pediatric + general), compared to 4.9% (+ 46.9%) of infants in Group 2.

Also, in the season 4 the proportion of RSV-coded admissions out of the total number of admissions for Group 1 plus Group 2 is higher in pediatric hospitals than in general hospitals (52.8% and 42.6%, respectively). This difference increases when considering monthly data: December 2018: 66.1% vs. 50.0%; January 2019: 65.6% vs. 53.1%; February 2019: 65.0% vs. 51.0%; March 2019: 61.1% vs. 40.1%).

The mean cost for hospitalization increased from season 1 to season 4 for Group 1 and Group 2: Group 1, from € 2,483 to € 2,617 (+ 5.4%); Group 2, from € 2,007 to € 2,180 (+ 8.6%). In season 4, the total costs for all infants hospitalized in Italy were: Group 1: € 20,203,406; Group 2: € 22,124,502.

## Discussion

Our results show that hospitalization rate of RSV-coded infants increased (+ 42.4%) from season 1 to season 4, compared to a smaller increase in Group 2 (+ 11.4%). The range of hospitalization rate for Group 1 was 125 per 10,000 infants in season 1 to 178 per 10,000 infants in season 4. These rates are higher than that of another Italian study (67 per 10,000 infants) [17] that used the same data source of our study, considering the years 2001–2014; this difference could be due to the fact that this study considered the same codes of our Group 1 but located only in primary position. Considering other studies conducted in other European countries, our results are in line with a Swedish study (174 per 10,000 infants) [18], but lower than a Spanish study (414 per 10,000 infants) [19]. However, this last study [19] also included codes for bronchitis and bronchiolitis, while in our study we considered bronchiolitis code in Group 2 (ICD-9 code 466.19). Another study conducted in UK [20] reported an incidence rate of bronchiolitis of 242 per 10,000 infants, of which only the 28% were RSV-coded, while the remainder were unspecified.

Our results showed that hospitalization burden was higher in RSV-coded infants than in infants without RSV code. In particular, the mean length of hospital stay for RSV-coded infants was statistically longer than that of non-RSV-coded infants, across all four seasons. The length of hospital stay for RSV-coded infants (6.3 days) was similar to that of other studies [17, 19, 21, 22], while Svensson et al. [18] (Sweden) and Murray et al. [20] (England) reported a median hospital stay of three days and 1 day, respectively. In season 4, most of RSV-coded infants (78.8%) required oxygen therapy or ventilation support, compared to 50.0% of non-RSV-coded. Our study showed that 33.2% of infants in Group1 and 18.5% in Group 2 required ventilation support. This percentage is much higher than that reported by Svensson et al. (6.3%) [18].

In our study, we estimated a mean cost for hospitalization for RSV-coded infants that ranged from € 2,483 (season 1) to € 2,617 (season 4). Another Italian study [22] conducted at Bambino Gesù Children Hospital (Rome) reported higher costs than ours: € 5,753 ± 2,042 for RSV infants, and € 5,395 ± 2,041 for infants with bronchiolitis due to other pathogens. However, this study in addition to DRG tariffs for hospitalizations, considered also the actual direct costs incurred for each patient, such as laboratory and imaging examinations, specialist evaluations, and therapies. Also, we may have underestimated the real costs because we did not consider indirect costs, such as time spent by parents caring for their child. On the other hand, a Spanish study [19] reported in the period 2002–2011 a mean hospitalization costs of € 2,166 in children with RSV bronchiolitis aged less than 2 years, which was similar to that calculated in our study.

As a strength of our study, we included all Italian infant population. Also, we included both principal and secondary diagnoses of RSV. We also acknowledged some limitations. First, the quality of routinely collected administrative data has not been validated against a reference standard. Second, although our assumption that Group 2 could contain a large portion of children affected by RSV is supported by clinical expertise and literature data, we could not verify the actual etiology of bronchiolitis. A reason why some of Group 2 children were not diagnosed with RSV is probably the lack of laboratory examinations during the hospital stay, due to unavailability or high costs of diagnostic tests. Consequently, aside from a possible increase in RSV spread in the study period, we could not exclude that the increase of hospitalization rate for Group 1 is related to a higher diagnostic accuracy during the hospital stay.

## Conclusions

Our results confirmed that RSV pulmonary disease in infants is seasonal and often requires hospitalization. Our study suggested that RSV is responsible for an increasing hospitalization rate and related costs during the study period.

## Electronic supplementary material

Below is the link to the electronic supplementary material.

Supplementary Material 1

## Data Availability

All data supporting the findings of this study are available within the paper and its supporting file.
