# Correction: Trends in hospitalizations of children with respiratory syncytial virus aged less than 1 year in Italy, from 2015 to 2019

**DOI:** 10.1186/s13052-024-01719-5

**Published:** 2024-09-03

**Authors:** Renato Cutrera, Daniela d’Angela, Massimiliano Orso, Liliana Guadagni, Anna Chiara Vittucci, Ilaria Bertoldi, Barbara Polistena, Federico Spandonaro, Ciro Carrieri, Eva Agostina Montuori, Raffaella Iantomasi, Luigi Orfeo

**Affiliations:** 1 Pediatric Pulmonology & Cystic Fibrosis Unit, Respiratory Research Unit, Bambino Gesù Children’s Hospital, IRCCS, Rome, Italy; 2 C.R.E.A. Sanità (Centre for Applied Economic Research in Healthcare), Rome, Italy; 3 University of Rome Tor Vergata, Rome, Italy; 4 Department of Surgical and Biomedical Sciences, University of Perugia, Perugia, Italy; 5 Hospital University Pediatrics Clinical Area, Bambino Gesù Children’s Hospital IRCCS, Rome, Italy; 6 Vaccine Medical Department, Pfizer, Rome, Italy; 7 Neonatal Intensive Care Unit, Ospedale Isola Tiberina Gemelli Isola, Rome, Italy

**Italian Journal of Pediatrics (2024) 50:119**

10.1186/s13052-024-01688-9

The original article mistakenly presented Figure 6 in Italian due to a missed correction by the production team that handled the article. The figure has since been translated to English.

**Fig. 6:**
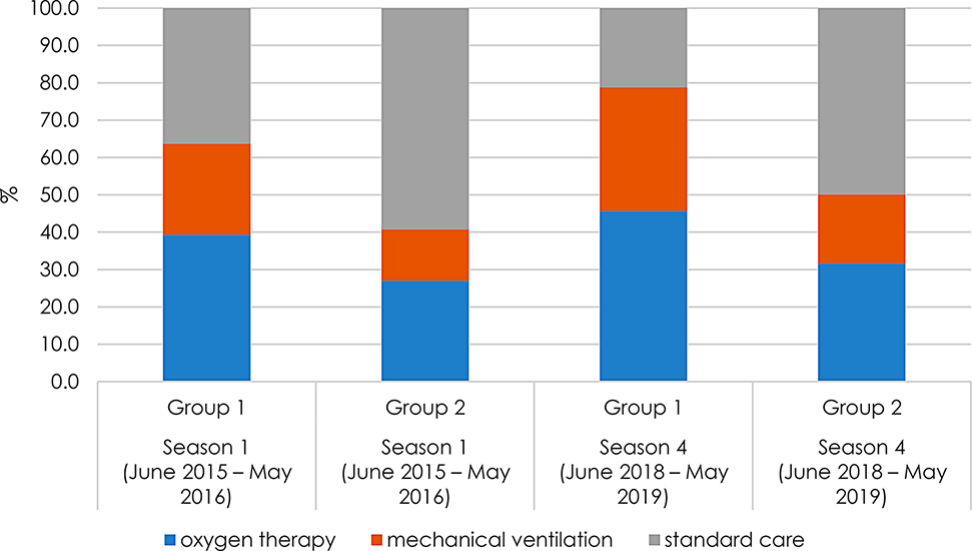
Rates of critical care cases out of all hospitalizations

