# Supplementary material for: Trends in hospitalizations of children with respiratory syncytial virus aged less than 1 year in Italy, from 2015 to 2019
Source: Ital J Pediatr. 2024 Jun 21;50:119. doi: 10.1186/s13052-024-01688-9 (PMC11191168; doi:10.1186/s13052-024-01688-9)
Supplement: Supplementary file 1 — Supplementary Material 1 [file 13052_2024_1688_MOESM1_ESM.docx]

Additional file 1. List of DRGs considered in season 4.

| **GROUP 1** | |
| --- | --- |
| **DRG code** | **DRG description** |
| 098 | BRONCHITIS & ASTHMA AGE 0-17 |
| 087 | PULMONARY EDEMA & RESPIRATORY FAILURE |
| 565 | RESPIRATORY SYSTEM DIAGNOSES WITH ASSISTED BREATHING MAJOR 96 HOURS |
| 386 | EXTREME IMMATURITY OR RESPIRATORY DISTRESS SYNDROME, NEONATE |
| 389 | FULL TERM NEONATE W MAJOR PROBLEMS |
| 566 | RESPIRATORY SYSTEM DIAGNOSES WITH LESS THAN 96 HOURS ASSISTED BREATHING |
| 100 | RESPIRATORY SIGNS & SYMPTOMS W/O CC |
| 091 | SIMPLE PNEUMONIA & PLEURISY AGE 0-17 |
| 081 | RESPIRATORY INFECTIONS & INFLAMMATIONS AGE 0-17 |
| 099 | RESPIRATORY SIGNS & SYMPTOMS W CC |
| 422 | VIRAL ILLNESS & FEVER OF UNKNOWN ORIGIN AGE 0-17 |
| 390 | NEONATE W OTHER SIGNIFICANT PROBLEMS |
| 541 | TRACH W MV 96+HRS OR PDX EXC FACE, MOUTH, & NECK DX W/MAJ OR |
| 387 | PREMATURITY W MAJOR PROBLEMS |
| 423 | OTHER INFECTIOUS & PARASITIC DISEASES DIAGNOSES |
| 385 | NEONATES, DIED OR TRANSFERRED TO ANOTHER ACUTE CARE FACILITY |
| 417 | SEPTICEMIA AGE 0-17 |
| 298 | NUTRITIONAL & MISC METABOLIC DISORDERS AGE 0-17 |
| 137 | CARDIAC CONGENITAL & VALVULAR DISORDERS AGE 0-17 |
| 101 | OTHER RESPIRATORY SYSTEM DIAGNOSES W CC |
| 316 | RENAL FAILURE |
| 108 | OTHER CARDIOTHORACIC PROCEDURES |
| 105 | CARDIAC VALVE & OTH MAJOR CARDIOTHORACIC PROC W/O CARD CATH |
| 388 | PREMATURITY W/O MAJOR PROBLEMS |
| 569 | MAJOR INTERVENTIONS ON LARGE AND SMALL INTESTINE WITH CC WITH MAJOR GASTROINTESTINAL DIAGNOSIS |
| 070 | OTITIS MEDIA & URI AGE 0-17 |
| 026 | SEIZURE & HEADACHE AGE 0-17 |
| 322 | KIDNEY & URINARY TRACT INFECTIONS AGE 0-17 |
| 102 | OTHER RESPIRATORY SYSTEM DIAGNOSES W/O CC |
| 127 | HEART FAILURE & SHOCK |
| 397 | COAGULATION DISORDERS |
| 574 | MAJOR HEMATOLOGIC/IMMUNOLOGIC DIAGNOSES EXCEPT SICKLE CELL ANEMIA AND COAGULOPATHIES |
| 468 | EXTENSIVE O.R. PROCEDURE UNRELATED TO PRINCIPAL DIAGNOSIS |
| 429 | ORGANIC DISTURBANCES & MENTAL RETARDATION |
| 144 | OTHER CIRCULATORY SYSTEM DIAGNOSES W CC |
| 147 | RECTAL RESECTION W/O CC |
| 398 | RETICULOENDOTHELIAL & IMMUNITY DISORDERS W CC |
| 149 | MAJOR SMALL & LARGE BOWEL PROCEDURES W/O CC |
| 572 | MAJOR GASTROINTESTINAL DISEASES AND PERITONEAL INFECTIONS |
| 175 | G.I. HEMORRHAGE W/O CC |
| 184 | ESOPHAGITIS, GASTROENT & MISC DIGEST DISORDERS AGE 0-17 |
| 190 | OTHER DIGESTIVE SYSTEM DIAGNOSES AGE 0-17 |
| 470 | UNGROUPABLE |
| 464 | SIGNS & SYMPTOMS W/O CC |
| 477 | NON-EXTENSIVE O.R. PROCEDURE UNRELATED TO PRINCIPAL DIAGNOSIS |
| 156 | STOMACH, ESOPHAGEAL & DUODENAL PROCEDURES AGE 0-17 |
| 017 | NONSPECIFIC CEREBROVASCULAR DISORDERS W/O CC |
| 396 | RED BLOOD CELL DISORDERS AGE 0-17 |
| 085 | PLEURAL EFFUSION W CC |
| 432 | OTHER MENTAL DISORDER DIAGNOSES |
| 139 | CARDIAC ARRHYTHMIA & CONDUCTION DISORDERS W/O CC |
| 341 | PENIS PROCEDURES |
| 467 | OTHER FACTORS INFLUENCING HEALTH STATUS |
| 034 | OTHER DISORDERS OF NERVOUS SYSTEM W CC |
| 241 | CONNECTIVE TISSUE DISORDERS W/O CC |
| 093 | INTERSTITIAL LUNG DISEASE W/O CC |
| 463 | SIGNS & SYMPTOMS W CC |
| 206 | DISORDERS OF LIVER EXCEPT MALIG, CIRR, ALC HEPA W/O CC |
| 021 | VIRAL MENINGITIS |
| 045 | NEUROLOGICAL EYE DISORDERS |
| 095 | PNEUMOTHORAX W/O CC |
| 181 | G.I. OBSTRUCTION W/O CC |
| 399 | RETICULOENDOTHELIAL & IMMUNITY DISORDERS W/O CC |
| 425 | ACUTE ADJUSTMENT REACTION & PSYCHOSOCIAL DYSFUNCTION |
| 333 | OTHER KIDNEY & URINARY TRACT DIAGNOSES AGE 0-17 |
| 446 | TRAUMATIC INJURY AGE 0-17 |
| 245 | BONE DISEASES & SPECIFIC ARTHROPATHIES W/O CC |
| 071 | LARYNGOTRACHEITIS |

| **GROUP 2** | |
| --- | --- |
| **DRG code** | **DRG description** |
| 098 | BRONCHITIS & ASTHMA AGE 0-17 |
| 087 | PULMONARY EDEMA & RESPIRATORY FAILURE |
| 386 | EXTREME IMMATURITY OR RESPIRATORY DISTRESS SYNDROME, NEONATE |
| 100 | RESPIRATORY SIGNS & SYMPTOMS W/O CC |
| 389 | FULL TERM NEONATE W MAJOR PROBLEMS |
| 565 | RESPIRATORY SYSTEM DIAGNOSES WITH ASSISTED BREATHING MAJOR 96 HOURS |
| 566 | RESPIRATORY SYSTEM DIAGNOSES WITH LESS THAN 96 HOURS ASSISTED BREATHING |
| 091 | SIMPLE PNEUMONIA & PLEURISY AGE 0-17 |
| 481 | BONE MARROW TRANSPLANT |
| 099 | RESPIRATORY SIGNS & SYMPTOMS W CC |
| 390 | NEONATE W OTHER SIGNIFICANT PROBLEMS |
| 422 | VIRAL ILLNESS & FEVER OF UNKNOWN ORIGIN AGE 0-17 |
| 387 | PREMATURITY W MAJOR PROBLEMS |
| 541 | TRACH W MV 96+HRS OR PDX EXC FACE, MOUTH, & NECK DX W/MAJ OR |
| 385 | NEONATES, DIED OR TRANSFERRED TO ANOTHER ACUTE CARE FACILITY |
| 423 | OTHER INFECTIOUS & PARASITIC DISEASES DIAGNOSES |
| 137 | CARDIAC CONGENITAL & VALVULAR DISORDERS AGE 0-17 |
| 081 | RESPIRATORY INFECTIONS & INFLAMMATIONS AGE 0-17 |
| 298 | NUTRITIONAL & MISC METABOLIC DISORDERS AGE 0-17 |
| 101 | OTHER RESPIRATORY SYSTEM DIAGNOSES W CC |
| 417 | SEPTICEMIA AGE 0-17 |
| 574 | MAJOR HEMATOLOGIC/IMMUNOLOGIC DIAGNOSES EXCEPT SICKLE CELL ANEMIA AND COAGULOPATHIES |
| 322 | KIDNEY & URINARY TRACT INFECTIONS AGE 0-17 |
| 102 | OTHER RESPIRATORY SYSTEM DIAGNOSES W/O CC |
| 464 | SIGNS & SYMPTOMS W/O CC |
| 105 | CARDIAC VALVE & OTH MAJOR CARDIOTHORACIC PROC W/O CARD CATH |
| 388 | PREMATURITY W/O MAJOR PROBLEMS |
| 075 | MAJOR CHEST PROCEDURES |
| 003 | CRANIOTOMY AGE 0-17 |
| 093 | INTERSTITIAL LUNG DISEASE W/O CC |
| 026 | SEIZURE & HEADACHE AGE 0-17 |
| 108 | OTHER CARDIOTHORACIC PROCEDURES |
| 110 | MAJOR CARDIOVASCULAR PROCEDURES W CC |
| 156 | STOMACH, ESOPHAGEAL & DUODENAL PROCEDURES AGE 0-17 |
| 184 | ESOPHAGITIS, GASTROENT & MISC DIGEST DISORDERS AGE 0-17 |
| 163 | HERNIA PROCEDURES AGE 0-17 |
| 127 | HEART FAILURE & SHOCK |
| 092 | INTERSTITIAL LUNG DISEASE W CC |
| 399 | RETICULOENDOTHELIAL & IMMUNITY DISORDERS W/O CC |
| 070 | OTITIS MEDIA & URI AGE 0-17 |
| 074 | OTHER EAR, NOSE, MOUTH & THROAT DIAGNOSES AGE 0-17 |
| 205 | DISORDERS OF LIVER EXCEPT MALIG, CIRR, ALC HEPA W CC |
| 303 | KIDNEY, URETER & MAJOR BLADDER PROCEDURES FOR NEOPLASM |
| 238 | OSTEOMYELITIS |
| 398 | RETICULOENDOTHELIAL & IMMUNITY DISORDERS W CC |
| 035 | OTHER DISORDERS OF NERVOUS SYSTEM W/O CC |
| 396 | RED BLOOD CELL DISORDERS AGE 0-17 |
| 408 | MYELOPROLIF DISORD OR POORLY DIFF NEOPL W OTHER O.R.PROC |
| 077 | OTHER RESP SYSTEM O.R. PROCEDURES W/O CC |
| 432 | OTHER MENTAL DISORDER DIAGNOSES |
| 145 | OTHER CIRCULATORY SYSTEM DIAGNOSES W/O CC |
| 095 | PNEUMOTHORAX W/O CC |
| 207 | DISORDERS OF THE BILIARY TRACT W CC |
| 441 | HAND PROCEDURES FOR INJURIES |
| 571 | MAJOR DISEASES OF THE ESOPHAGUS |
| 034 | OTHER DISORDERS OF NERVOUS SYSTEM W CC |
| 174 | G.I. HEMORRHAGE W CC |
| 190 | OTHER DIGESTIVE SYSTEM DIAGNOSES AGE 0-17 |
| 030 | TRAUMATIC STUPOR & COMA, COMA <1 HR AGE 0-17 |
| 463 | SIGNS & SYMPTOMS W CC |
| 086 | PLEURAL EFFUSION W/O CC |
| 429 | ORGANIC DISTURBANCES & MENTAL RETARDATION |
| 138 | CARDIAC ARRHYTHMIA & CONDUCTION DISORDERS W CC |
| 467 | OTHER FACTORS INFLUENCING HEALTH STATUS |
| 139 | CARDIAC ARRHYTHMIA & CONDUCTION DISORDERS W/O CC |
| 404 | LYMPHOMA & NON-ACUTE LEUKEMIA W/O CC |
| 247 | SIGNS & SYMPTOMS OF MUSCULOSKELETAL SYSTEM & CONN TISSUE |
| 181 | G.I. OBSTRUCTION W/O CC |
| 088 | CHRONIC OBSTRUCTIVE PULMONARY DISEASE |
| 284 | MINOR SKIN DISORDERS W/O CC |
| 206 | DISORDERS OF LIVER EXCEPT MALIG, CIRR, ALC HEPA W/O CC |
| 340 | TESTES PROCEDURES, NON-MALIGNANCY AGE 0-17 |
| 071 | LARYNGOTRACHEITIS |
| 270 | OTHER SKIN, SUBCUT TISS & BREAST PROC W/O CC |
| 431 | CHILDHOOD MENTAL DISORDERS |
| 451 | POISONING & TOXIC EFFECTS OF DRUGS AGE 0-17 |
| 301 | ENDOCRINE DISORDERS W/O CC |
| 282 | TRAUMA TO THE SKIN, SUBCUT TISS & BREAST AGE 0-17 |
